# The Physician’s Visiting List for 1888

**Published:** 1887-11

**Authors:** 


**The Physician's Visiting List for 1888.** Thirty-seventh year of its publication. Philadelphia: P. Blakiston, Son & Co., 1,012 Walnut street.

The receipt of this list reminds us that another year is almost upon us. The "old favorite" is usually the first in the field, and will, in a few weeks, be followed by a score of others, but in professional favor, "Lindsay & Blakiston's List" still holds first place, and bids fair to retain that position for a long time to come. The publishers spare no efforts to make it the best list in the market.

